# Mesoporous Silica Administration as a New Strategy in the Management of Warfarin Toxicity: An In-Vitro and In-Vivo Study

**DOI:** 10.34172/apb.42665

**Published:** 2024-10-02

**Authors:** Fatemeh Farjadian, Fatemeh Parsi, Reza Heidari, Khatereh Zarkesh, Hamid Reza Mohammadi, Soliman Mohammadi-Samani, Lobat Tayebi

**Affiliations:** ^1^Pharmaceutical Sciences Research Center, Faculty of Pharmacy, Shiraz University of Medical Sciences, Shiraz, Iran.; ^2^Department of Pharmaceutics, Faculty of Pharmacy, Shiraz University of Medical Sciences, Shiraz, Iran.; ^3^Department of Pharmaceutics, Faculty of Pharmacy, Hormozgan University of Medical Sciences, Bandar Abbas, Iran.; ^4^Department of Toxicology, School of Pharmacy, Lorestan University of Medical Sciences, Khorramabad, Iran.; ^5^School of Dentistry, Marquette University, Milwaukee, USA.; ^6^Institute for Engineering in Medicine, Health & Human Performance (EnMed), Batten College of Engineering and Technology, Old Dominion University, Norfolk, VA, USA.

**Keywords:** Adsorbent, Antidote, Mesoporous silica, Warfarin, In vivo, In vitro

## Abstract

**Purpose:**

Warfarin is one of the most widely used anticoagulants that functions by inhibiting vitamin K epoxide reductase. Warfarin overdose, whether intentional or unintentional, can cause life-threatening bleeding. Here, we present a novel warfarin adsorbent based on mesoporous silica that could serve as an antidote to warfarin toxicity.

**Methods:**

Amino-functionalized mesoporous silica (MS-NH_2_) was synthesized based on the co-condensation method through a soft template technique followed by template removal. The prepared structure and functional group were studied by Fourier transform infrared spectroscopy (FT-IR), and X-ray diffraction (XRD). Scanning electron microscopy (SEM) and transmission electron microscopy (TEM) checked the morphology. The capacity of MS-NH_2_ in the adsorption of warfarin was evaluated in vitro, at pH=7.4 and pH=1.2. In vivo evaluations were performed in control and warfarin-overdosed animal models. Overdosed animals were treated with MS-NH_2_ by oral gavage. Biomarkers of organ injury were assessed in animal serum.

**Results:**

The MS-NH_2_ was relatively uniform, spherical with defined diameters (400 nm) and porous structure. Synthesized particles had a large surface area (1015 m_2_ g^-1^) and mean pore diameter of 2.4 nm which led to considerable adsorption capacity for warfarin 1666 mg/g. In vivo studies revealed that oral administration of MS-NH_2_ in mice poisoned with warfarin caused a significant difference (*P*<0.05) in the International Normalized Ratio (INR) and prothrombin time (PT). Moreover, the warfarin with MS-NH_2_ group demonstrated a notable decrease in biomarkers associated with tissue damage, such as bilirubin, lactate dehydrogenase (LDH), alanine aminotransferase (ALT), and aspartate aminotransferase (AST).

**Conclusion:**

The results confirm that MS-NH_2_ administration can be an effective treatment for warfarin toxicity and could potentially mitigate the adverse effects of warfarin poisoning.

## Introduction

 Warfarin is one of the most recommended and commonly utilized anticoagulants, which reduces the body’s ability to form blood clots in various thromboembolic conditions. This drug also plays an essential role in preventing clot formation and facilitating blood circulation.^1^ By limiting the activity of vitamin K, warfarin inhibits clotting factors that require vitamin K, including clotting factors II, VII, IX, X, and proteins C and S. However, the dose of warfarin varies widely from person to person and depends on the rate of metabolism. The plasma concentration of warfarin is critical for its therapeutic effect.^2^ Where the level of warfarin in the blood is low, there is a risk of blood clots. On the other hand, if the level of this drug in the blood is increased, the possibility of bleeding increases.^3^ It is therefore important to monitor the concentration of this drug. The prothrombin time (PT) is used to measure the efficiency of warfarin administration. Another standard indicator of warfarin levels is the International Normalized Ratio (INR), which should be between 2 and 3. Patients with higher therapeutic levels are at risk of bleeding, while patients with an INR below this level require a higher dose of warfarin to protect against thromboembolic events.^4^ In addition, rapid absorption from the gastrointestinal tract and high protein binding can lead to toxicity and bleeding.^5,6^ Warfarin toxicity is common and occurs when the INR is greater than 4. ^7^ Oral and intravenous administration of vitamin K can reverse the anticoagulant effect within 24 and 4 to 8 hours, respectively.^8,9^ If there is a risk of life-threatening bleeding, such as hemorrhagic and hypovolemic shock and gastrointestinal bleeding, intravenous administration of fresh frozen plasma (FFP) or concentrated prothrombin is recommended.^10^ Anticoagulants were administrated to lower the risk of thrombosis in COVID-19.^11^

 Mesoporous silica nanoparticles (MSN) have been under consideration due to their high surface area to volume ratio, low toxicity, biocompatibility, and ability to bind to different functional groups in comparison to other types of nanoparticles. MSN has been used for various purposes including separation as a sensor, drug carrier, and drug adsorbent, as well as imaging and selective adsorption.^12,13^ Vallet-Regi et al have pioneered the use of MSN according to the MCM-41 (Mobil Composition of Matter-41) pattern to deliver drugs.^14,15^ Today, intelligent delivery systems made of MSNs, capable of responding to external and internal stimuli, are widely used for passive and active targeting of therapeutic agents, especially anti-cancer drugs and also for theranostic.^16-19^

 The development of a definitive antidote for the rapid treatment of warfarin would be a life-saving strategy. High surface area structures are effective adsorbents for toxins. In 2015, Farjadian et al revealed that mesoporous silica could be a unique and superior alternative to activated charcoal in the treatment of drug overdose.^20^ Furthermore, this group showed that EDTA-modified MSN could be a supreme adsorbent of iron and copper and could be applied as an antidote in *in vivo* models.^21,22^ They also showed that mesoporous silica could be an excellent adsorbent for ammonium and an antidote in the treatment of hepatic encephalopathy, and rice tablets in the control of phosphine poisoning in *in vivo* models.^23^ It was also demonstrated that sevelamer could be potent antidote of phosphine poisoning.^24^ Diatom was also introduced as an efficient biological antidote against iron overload.^25^ The other *In vitro* and *in vivo* study showed oral administration of mesoporous silica could be used as an adsorbent and antidote agent of methotrexate (MTX).^26^

 Following on from our previous studies, amine-modified mesoporous silica (MS-NH_2_) was synthesized and then identified. The efficiency of this structure in adsorbing warfarin was evaluated. Following on from our previous studies, MS-NH_2_ was synthesized and analyzed. The efficiency of this structure in adsorbing warfarin was evaluated in *in vitro* studies. In addition, *in vivo,* studies were performed in animal models to demonstrate the performance of MS-NH_2_ in reversing the symptoms of warfarin toxicity. The results were compared with vitamin K administration as standard treatment. Evaluations were based on serum biomarkers, PT, and INR levels for organ damage and histopathological observations in *in vitro* studies. In addition, *in vivo,* studies were performed in animal models to demonstrate the performance of MS-NH_2_ in alleviating the symptoms of warfarin toxicity. The results were compared with vitamin K administration as standard treatment. Evaluations were based on serum biomarkers, PT and INR levels for organ damage, and histopathological observations.

## Methods

###  Materials

 (3-aminopropyl) triethoxysilane (APTES), tetraethoxysilane (TEOS), trichloroacetic acid (TCA), ethanol, formaldehyde, and thiopental were purchased from a domestic supplier of Merck Company (Germany). Cetyl trimethylammonium bromide (CTAB) and 4-Hydroxy-3-(3-oxo-1-phenylbutyl) coumarin (warfarin) was supplied from Sigma-Aldrich. An acetone and ammonia solution of 25% was provided by Kimia Mavad (Iran). Hydrochloric acid (HCl) was obtained from Alvand Chem (Iran). Phosphate-buffered saline (PBS) was provided from Atocel (Austria).

###  Instruments

 Various techniques and apparatus were used to evaluate the chemical properties. Fourier transform infrared spectroscopy (FT-IR) (Bruker) was performed to identify the functional groups in the spectral range of 400-4000 cm^-1^. Energy dispersive X-ray spectroscopy (EDX) was performed to find elements in the sample. X-ray diffraction (XRD) (MPD 3000) was carried out to characterize the crystallography of the structure. The surface properties and porosity of the nanoparticles were calculated with a nitrogen adsorption and desorption apparatus and various parameters were obtained by the Brunauer-Emmett-Teller (BET) theory (Micrometrics Traitor II Plus). The hydrodynamic dimensions and size distribution of the particles were assessed using dynamic light scattering (Microtrac USA). The zeta potential was used to measure the surface charge of the nanoparticles (Nanotrac Wave MN402 (USA)). The size of the particles and morphology were analyzed with field emission scanning electron microscopy (FE-SEM) (Mira III- TeScan) and High-resolution transmission electron microscopy (HRTEM) (JEOl JEM 2010). In addition, UV-Vis spectroscopy (T92plus) was used to analyze the warfarin content in different pharmaceutical matrices.

###  Preparation of amino-functionalized mesoporous silica (MS-NH_2_)

 First, 6.6 mmol CTAB was dissolved as a templating agent in 50 ml DW and 50 mL ethanol. The solution was then mixed with 13 ml of 25% ammonia and stirred vigorously (30 min). Then 14.8 mmol TEOS and 1.6 mmol APTES were added to the mixture. The mixture was stirred at 45 ℃ for 2 hours. The white mixture was filtered and washed with deionized water, three times with ethanol and acetone. Mesoporous silica (MS) with amine functional groups was dried in an oven at 60 °C for 24 hours. In the next step, to remove the templating agent (CTAB) and for further purification, 200 mL of ethanol and 3 mL of hydrochloric acid were added to the white powder and refluxed (48 hours at 80 °C).^27^ The mixture was passed through a Buchner flask and washed twice with ethanol and acetone. Finally, MS-NH_2_ was collected and dried in an oven at 50 °C.

###  Warfarin calibration curve

 Warfarin stock solution was prepared at a concentration of 20 µg /mL in a medium containing PBS medium with pH 7.4 and an acidic solution of hydrochloric acid with pH 1.5. Standard samples with concentrations of 2, 4, 6, 8, 12, and 16 µg /mL were prepared using the serial dilution technique. The absorbance of the solutions was then analyzed by UV-Vis spectrometry at 308 nm.^28^

###  Adsorption study

 To evaluate the quantity of warfarin absorption with MS-NH_2_, samples with different ratios of 0.2, 0.5, 1, 2, and 5 warfarin (mmol) /NH_2_ (mmol) of MS-NH_2_ were added to the appropriate vessel containing suitable buffer at pH 7.2 and 1.2. After 2 hours, the mixture was centrifuged and the absorbance of the supernatants was read with a UV-Vis spectrophotometer at a wavelength of 308 nm. All adsorption tests were repeated three times. The adsorption capacity of the drug (q) (mmol/g) was then assessed using the following equation (equation 1).^29^


Eq. 1 $$q=\left(C_{0}-C_{e}\right)\;V/m$$

where C_e_ is the equilibrium concentration of warfarin (mg/L, C_0_ is the primary concentration of warfarin and V is the sample volume (L).

###  Isothermal absorption models

 Two distinct models, the Freundlich and Langmuir isotherms, were used to assess the adsorption capability of warfarin on MS-NH2. While the Freundlich model illustrates adsorption in heterogeneous systems, the Langmuir isotherm is frequently employed to characterize the adsorption profile in a homogeneous matrix with monolayer adsorption. The following equation is used to illustrate the linear Langmuir model (equations 2 & 3).^30^


Eq. 2 $$C_{e}/q_{e}=1/q_{m}\times C_{e}+1/k_{a}q_{m}$$

Eq. 3 $$1/q_{e}=\left[1/k_{a}q_{m}\right]\times 1/C_{e}+1/q_{m}$$

In this equation, q_e_ is the amount of warfarin adsorbed on MS-NH_2_ (mmol/g), k_a_ is the experimental adsorption constant and q_m_ is the maximum adsorption rate. C_e_ is the equilibrium concentration of warfarin (mg/L). The Freundlich linear equation is described here (equation 4).


Eq. 4 $$q_{e}=k_{f}C_{e}^{1/n}$$

K_f_ is the adsorption constant of Freundlich, and 1/n is the adsorption intensity.^31^

###  Animal welfare

 25 Male BALB/C mice (20-30 g) were supplied from Shiraz University of Medical Sciences. All experiments were performed by the Iran National Committee for Ethics in Biomedical Research and acquired an Ethical allowance (IR.SUMS.REC.1400.263).

###  In vivo studies

 Animals (mice) were randomly divided into five groups. The study groups were selected in the following order.

1. The control group received only normal saline (to dissolve warfarin).
2. The group receiving only 5 mg/kg warfarin orally (by gavage).^32^
3. The group receiving 5 mg/kg warfarin orally (by gavage) + 1 mg/kg mesoporous silica compounds (MS-NH_2_) (by gavage).
4. The group that received 5 mg/kg warfarin orally (gavage) + 10 mg/kg mesoporous silica compounds (MS-NH_2_) orally (gavage).
5. The group received 5 mg/kg warfarin orally (gavage) + vitamin K (0.5 mg/kg) by injection.

1 mL blood was collected from the abdominal aorta and the serum of the samples was separated by centrifugation at 1000 rpm for 25 minutes. The collected serum was kept at -20 °C for further experiments. In addition, PT and INR were measured for warfarin toxicity. In this project, 4% sodium citrate was used as the anticoagulant (100 μL of sodium citrate per 1 mL of blood), and blood clotting time was defined as PT.^33^

 In addition, markers of liver, kidney, and heart tissue damage, including bilirubin, lactate dehydrogenase (LDH),^34^ Aspartate aminotransferase (AST) and Alanine aminotransferase (ALT), levels,^35^ were calculated in animal serum by commercial kits.

###  Histological analysis

 The animals were sacrificed. The kidneys, liver, and heart of the mice were removed. After washing with normal saline, the organs were fixed in 10% formalin. The organ sections were stained with haematoxylin-eosin and examined microscopically.^36^

###  Statistical analysis

 One-way analysis of variance (ANOVA), Tukey’s post hoc test, and the Student’s t-test were used to evaluate the data (SPSS, version 21.0, IBM, New York, USA). Results were represented as SEM ± mean and were deemed significant at *P*< 0.05. Graphs were plotted using SigmaPlot 12.5.

## Results and Discussion

###  Preparation and physicochemical properties of mesoporous silica (MS-NH_2_)

 In the present study, MS-NH_2_ nanoparticles were synthesized by two-step co-condensation using CTAB as the templating agent, TEOS as the main substrate, and APTES as the coupling agent. FTIR spectroscopy was used to evaluate the chemical modification (Figure S1). The peak at 1680 is attributed to C-N stretching, confirming the formation of the MS-NH_2_ structure. the band at 1100 cm^-1^ is characteristic of the Si-O-Si groups. In addition, the broad band at 3000-3800 cm^-1^ was attributed to hydroxyl groups interfering with the characteristic N-H band.^37^

 Elemental analysis showed that the weight percentages of nitrogen, carbon, and hydrogen were 3.01, 9.98, and 2.97%, respectively. The amount of organic layer (propylamine) was calculated to be 2.2 mmol/g.

 Low-angle X-ray diffraction was performed to show the crystallinity of the structure (Figure S2). A sharp peak appeared at 0.7 to 2° 2theta angles, characteristic of diffraction in the (100) xyz direction. A shorter broad band was observed at around 2.5° 2theta, corresponding to a diffraction index of 110. The overall structural observation depicted that the synthesized structure is crystalline with a regular structure following the diffraction model of mesoporous silica.^38^

**Figure 1:**
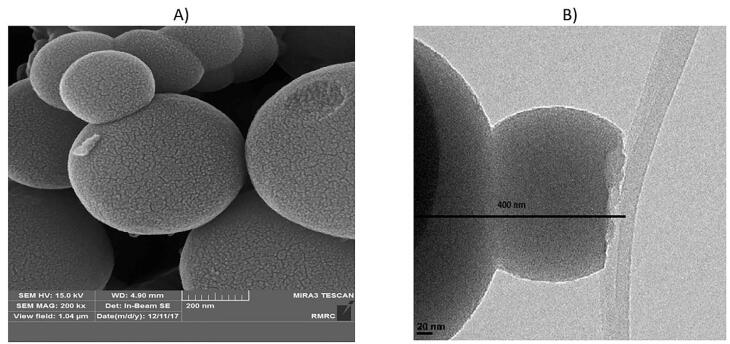
FESEM and HR-TEM images of MS-NH2

 FESEM and HR-TEM images were also employed to observe the morphology and size of the nanoparticles. The size of individual particles was around 400 nm (Figure 1A). The synthesized particles were relatively uniform and spherical with defined diameters, regular structures, and homogeneous shapes. The HR-TEM image (Figure 1B) demonstrated the existence of a regular porous structure with a particle size of nearly 400 nm, indicating that the particle structure is well formed.

 The hydrodynamic diameter of the nanoparticles in PBS was determined by light scattering. The maximum frequency of the particle size was 434 nm (Figure S3).

 The pore volume, pore diameter, and surface area were evaluated by N_2_ adsorption-desorption technique and the results were given as a summary in Table 1. According to the BET evaluation, the synthesized nanoparticles had a large surface area (1015 m^2^ g^-1^). Moreover, the considerable surface area and pore volume of MS-NH_2_ are particularly associated with higher warfarin absorption capacity.

**Table 1:** Surface area by BET, pore diameter, and pore volume of MS-NH_2_

**Nanoparticle**	**surface area** **(m^2^ g**^-1^**)**	**Mean pore** **diameter (nm)**	**pore volume** **(m^3^ g**^-1^**)**
MS-NH_2_	1015	2.7	0.67

###  Adsorption Study

 To measure the capacity of MS-NH_2_ in warfarin adsorption, several samples containing different concentrations of warfarin and fixed amounts of MS-NH_2_ were prepared. To determine the amount of warfarin in equilibrium concentration, standard calibration curves of warfarin in mediums with 2 different pHs (7.4 & 1.2) were considered.

**Figure 2:**
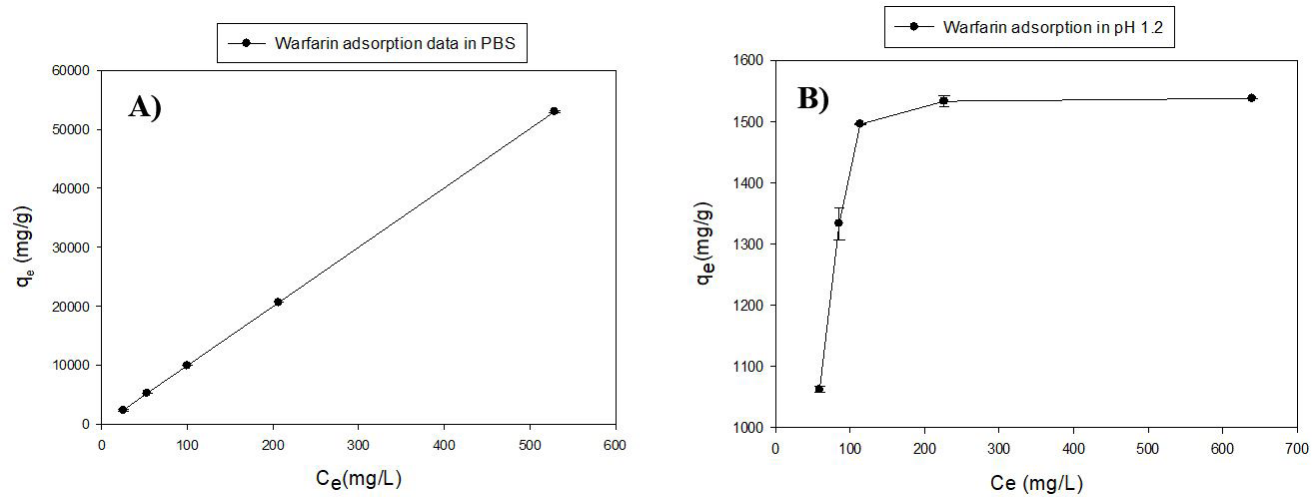
Adsorption diagrams of warfarin by MS-NH_2_. A) medium with pH 7.4 (PBS); (B) pH 1.2

 Adsorption experimentswere conducted at the simulated pH of the intestine and stomach (pH 7.4 (PBS) and 1.2, respectively). According to the results (Figure 2A), the ascending graph with a high slope indicates excellent adsorption of warfarin by MS-NH_2_ in two mediums. The MS-NH_2_ is able to form a hydrogen bond with warfarin at pH = 7.4; therefore, the graph did not reach the saturation limit at the concentration limit of this study,^39^ but in the acidic medium containing HCl (Figure 2B), the slope of the graph descended and reached a plateau level which shows that the absorption rate has reached its maximum with increasing concentration. This problem was due to amine protonation on the surface of MS-NH_2_, which reduced the hydrogen bond and, in this respect, was not able to adsorb more warfarin on its surface.^40^

**Figure 3:**
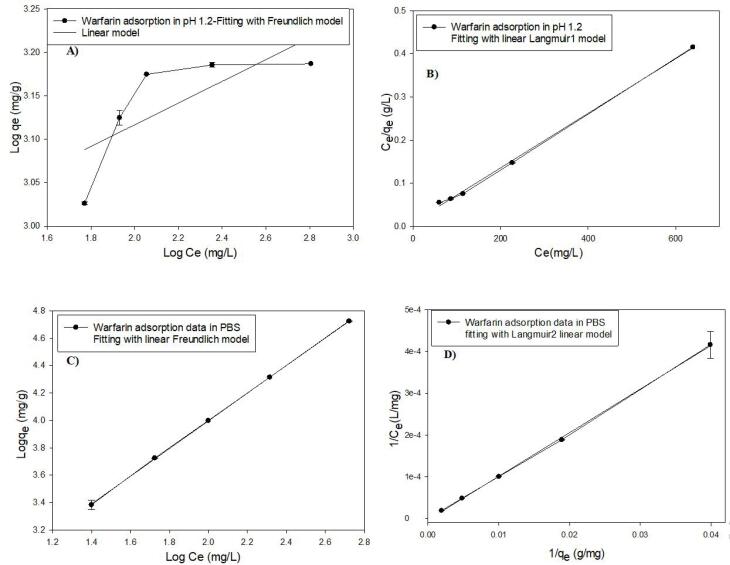
Fitting of warfarin adsorption data with linear models of Langmuir and Freundlich isotherm models. A) Fitting with linear equation of Freundlich model (Eq. 3) in pH 1.2, B) Fitting with linear equation (Eq. 2) of Langmuir model in pH 1.2, C) Fitting with linear equation of Freundlich model in pH 7.4, D) Fitting with linear equation (Eq. 2) of Langmuir model in pH 7.4

 To calculate the adsorption parameters such as adsorption constant and adsorption capacity, the plots (Figure 3A&B) were fitted with Langmuir and Freundlich linear isotherm models (Eq. 2 & 3). The fitting plots are shown in Figure 3 and the parameters are given in Table 2.

**Table 2:** Adsorption isotherms of warfarin onto MS-NH_2_ in pHs 1.2 and 7.4

**pH**	**Freundlich parameters**			**Langmuir parameters**		
	**n**	**K**_f_	**R**^2^	**q**_m_	**K**_1_	**R**^2^
1.2	7.72	713.12	0.59	1666	102.041	0.99
7.4	1.02	110.82	0.99	96	0.035	0.99

 The best-fitting adsorption model according to the correlation coefficient (R^2^) was the Langmuir model due to the protonation of the amine group at pH 1.2.^41^ Furthermore, the maximum adsorption capacity (q_m_) of warfarin by MSN-NH_2_ was 1666 mg/g. However, the absorption data followed both Langmuir and Freundlich models at neutral pH with R^2^ 0.99,^42^ indicating a monolayer absorption with different functional groups.^43^

###  In vivo studies

**Figure 4:**
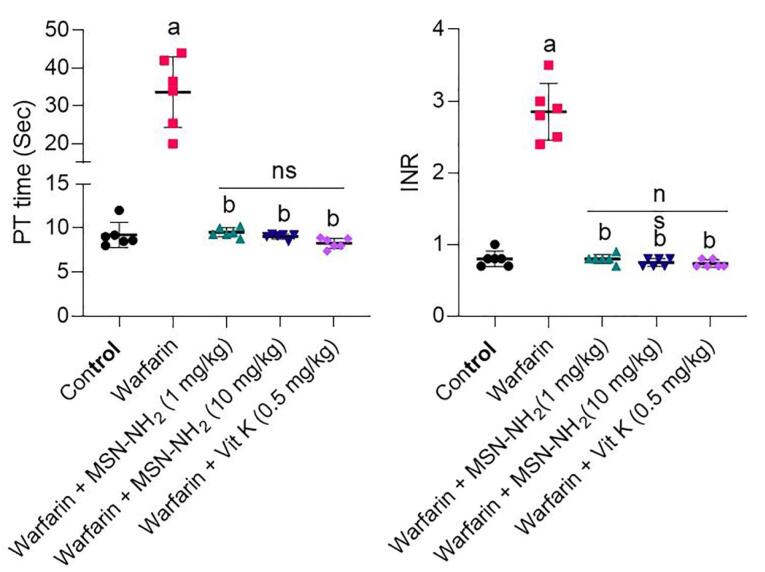
PT and INR in control groups (Black circle), warfarin (Red square, warfarin + MS-NH_2 _(1 mg/kg) (Green triangle, (warfarin + MS-NH_2_ (10 mg/kg) (Blue triangle) and warfarin + Vit K (0.5 mg/kg). Data are displayed as mean ± SD (n = 6). (a): Significant difference compared to the warfarin group; (b): Significant difference compared to the group treated with warfarin; ns: Not significant

 As mentioned above, PT and INR were chosen as toxicity indicators for warfarin. After administration of warfarin, warfarin plus MS-NH_2_, and warfarin with vitamin K in animal models, INR and PT were measured and compared. As shown in Figure 4, INR increased significantly in the warfarin group compared with the control group (*P* < 0.001). The warfarin-overdosed groups were treated with different doses of MS-NH_2_ (1 and 10 mg/kg) and vitamin K. When comparing warfarin-overdosed and MS-NH_2_ -treated animals, the former group showed a significant decrease in INR. Furthermore, no significant differences were observed between MS-NH_2_ treated animals at different doses (1 & 10 mg/kg) and vitamin K. PT results showed that in the warfarin group, PT increased significantly compared to the control group. As a result, the blood clotting time was remarkably reduced in animals receiving warfarin with MS-NH_2_ compared to the warfarin group. A significant reduction in PT was observed in the warfarin group with different doses of MS-NH_2_ (1 and 10 mg/kg) and in the warfarin group with vitamin K. It is noted that injected high-dose vitamin K can reduce PT and INR within 48 to 72 hours; however, the oral form MS-NH_2_ reduces these parameters within the first 24 hours of warfarin toxicity.^44^

**Figure 5:**
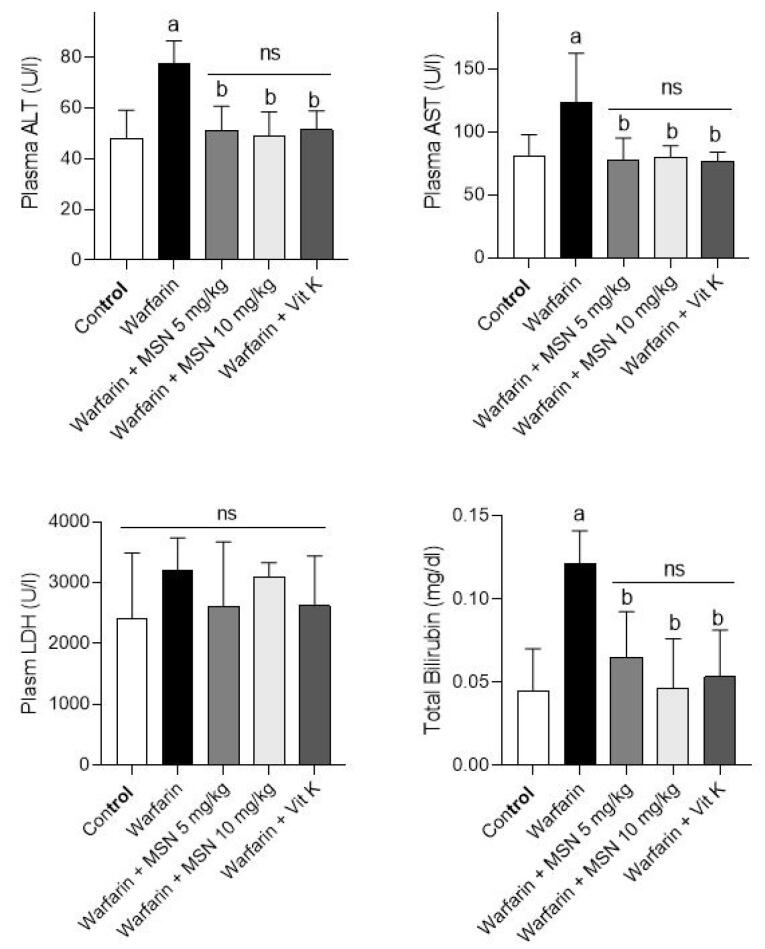
Serum biochemical measurements in warfarin-intoxicated animals. Data are represented as mean ± SD (n = 6). a: Notable difference from the control group (*P* < 0.001). b: Notable difference from the group receiving warfarin treatment (*P* < 0.001). ns: not significant

 Markers of damage to various tissues, including liver, kidney, heart, ALT, AST, bilirubin, and LDH levels were examined in the serum of animals in the above groups. The results are shown in Figure 5. The results of these biomarkers indicated that the plasma levels of AST, ALT, and total bilirubin were remarkably increased in the warfarin group compared to the normal saline group.^45^ These biomarkers significantly decreased in the warfarin groups with different doses of MS-NH_2_ (1 and 10 mg/kg) which lowered liver damage in warfarin toxicity. The results for LDH levels showed no differences between any of the groups compared with the control group and the warfarin-only group.

**Figure 6:**
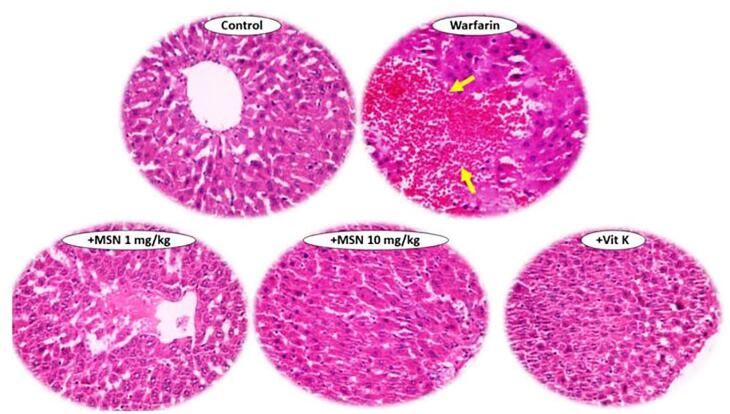
Histopathological examinations in the control, vitamin K, and warfarin group treated with 2 doses of 1 &10 mg/kg of MS-NH_2_

 Histopathology was performed on the liver, heart, and kidney; the data are shown in Figure 6. The results showed significant hemorrhaging (yellow arrow) in the liver tissue of animals treated with warfarin.^46^ There was also no warfarin-induced bleeding in liver tissue in the groups receiving warfarin with different doses of MS-NH_2_ and vitamin K. The results of this study suggest that oral MS-NH_2_ may be capable of significantly reducing the haemorrhagic effects and clotting time of warfarin toxicity. However, no significant histopathological altercations were detected in the heart, or kidneys of animals treated with warfarin.^47^

## Conclusion

 In this project, MS-NH_2_ was introduced as an effective antidote for warfarin overdose. morphological studies showed uniform and spherical mesoporous nanoparticles with defined diameters, regular structures, and homogeneous shapes. The BET analysis showed a high surface area of about 1015 m^2^ g^-1^. The warfarin adsorption followed the Langmuir model with a high adsorption capacity of 1666 mg/g at pH 1.2. The in vivo studies demonstrated that oral administration of MS-NH_2_ with warfarin could reduce INR, PT, and liver enzyme levels including AST, ALT, and total bilirubin. Histopathological studies showed no accumulation and damage of nanoparticles in vital organs. Overall, these amine-functionalized MSN nanoparticles can be proposed as a suitable antidote for the treatment of warfarin poisoning.

## Competing Interests

 None declared.

## Ethical Approval

 Animals were handled according to the Shiraz University of Medical Sciences guidelines for care and use of laboratory animals approved by a local ethics committee at Shiraz University of Medical Sciences, Shiraz, Iran (IR.SUMS.REC.1400.263)

## Supplementary Files

Supplementary file contains Figures S1-S3.
