# Supplementary material for: Mesoporous Silica Administration as a New Strategy in the Management of Warfarin Toxicity: An In-Vitro and In-Vivo Study
Source: Adv Pharm Bull. 2024 Oct 2;14(4):883–91. doi: 10.34172/apb.42665 (PMC11970487; doi:10.34172/apb.42665)
Supplement: Supplementary file 1 — Supplementary file contains Figures S1-S3. [file apb-14-883-s001.pdf]

Adv Pharm Bull. 2024;14(4):883-S1
doi: 10.34172/apb.42665
https://apb.tbzmed.ac.ir

Supplementary File

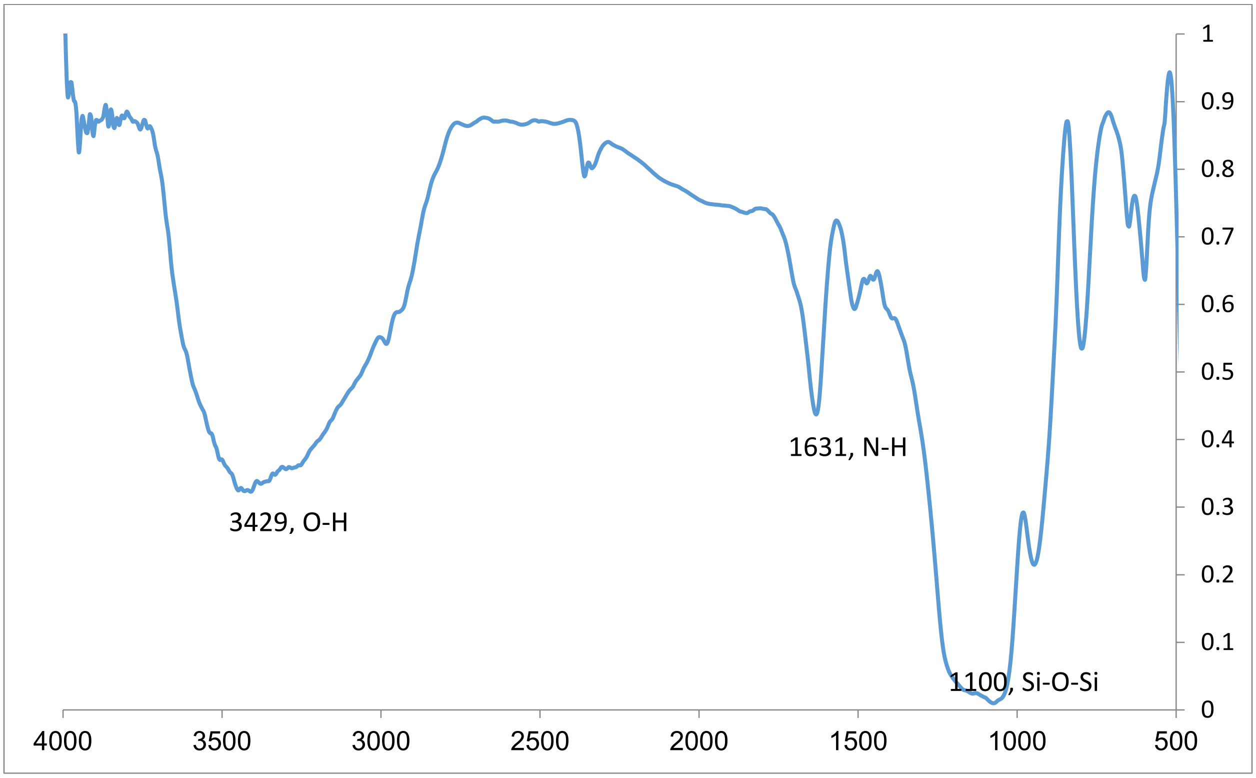

Figure S1. FT-IR spectrum of MSN-NH_2

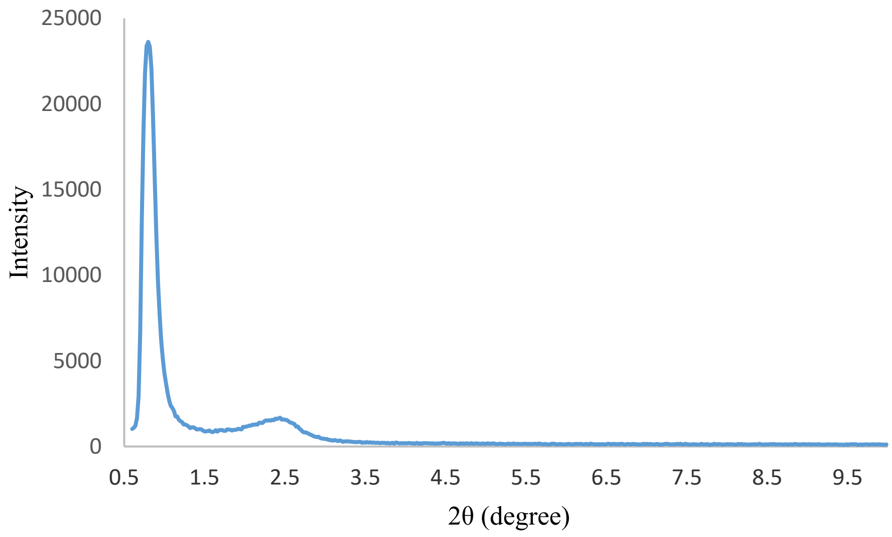

Figure S2. XRD patterns of $MS-NH_2$

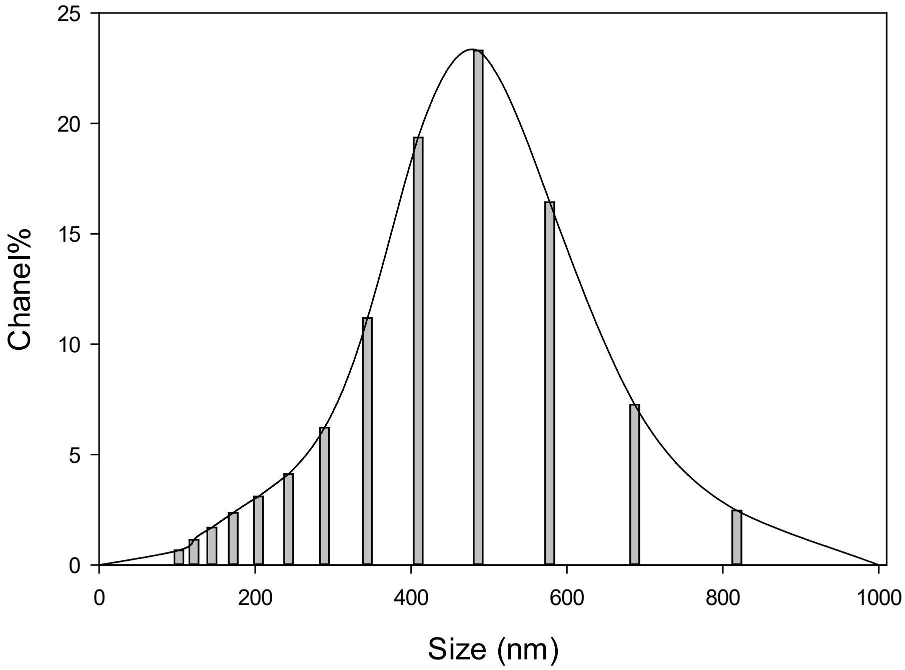

Figure S3. Dynamic light scattering, size distrubution histogram, $MS-NH_2$
